# Is a 4-Bit Synaptic Weight Resolution Enough? – Constraints on Enabling Spike-Timing Dependent Plasticity in Neuromorphic Hardware

**DOI:** 10.3389/fnins.2012.00090

**Published:** 2012-07-17

**Authors:** Thomas Pfeil, Tobias C. Potjans, Sven Schrader, Wiebke Potjans, Johannes Schemmel, Markus Diesmann, Karlheinz Meier

**Affiliations:** ^1^Kirchhoff Institute for Physics, Ruprecht-Karls-University HeidelbergHeidelberg, Germany; ^2^Computational and Systems Neuroscience (INM-6), Institute of Neuroscience and Medicine, Research Center JülichJülich, Germany; ^3^Brain and Neural Systems Team, RIKEN Computational Science Research ProgramWako-shi, Japan; ^4^RIKEN Brain Science InstituteWako-shi, Japan; ^5^RWTH Aachen UniversityAachen, Germany

**Keywords:** neuromorphic hardware, wafer-scale integration, large-scale spiking neural networks, spike-timing dependent plasticity, synaptic weight resolution, circuit variations, PyNN, NEST

## Abstract

Large-scale neuromorphic hardware systems typically bear the trade-off between detail level and required chip resources. Especially when implementing spike-timing dependent plasticity, reduction in resources leads to limitations as compared to floating point precision. By design, a natural modification that saves resources would be reducing synaptic weight resolution. In this study, we give an estimate for the impact of synaptic weight discretization on different levels, ranging from random walks of individual weights to computer simulations of spiking neural networks. The FACETS wafer-scale hardware system offers a 4-bit resolution of synaptic weights, which is shown to be sufficient within the scope of our network benchmark. Our findings indicate that increasing the resolution may not even be useful in light of further restrictions of customized mixed-signal synapses. In addition, variations due to production imperfections are investigated and shown to be uncritical in the context of the presented study. Our results represent a general framework for setting up and configuring hardware-constrained synapses. We suggest how weight discretization could be considered for other backends dedicated to large-scale simulations. Thus, our proposition of a *good hardware verification practice* may rise synergy effects between hardware developers and neuroscientists.

## 1 Introduction

Computer simulations have become an important tool to study cortical networks (e.g. Markram et al., 1997; Brunel, 2000; Morrison et al., 2005, 2007; Brette et al., 2007; Johansson and Lansner, 2007; Vogelstein et al., 2008; Kunkel et al., 2011; Yger et al., 2011). While they provide insight into activity dynamics that can not otherwise be measured *in vivo* or calculated analytically, their computation times can be very time-consuming and consequently unsuitable for statistical analyses, especially for learning neural networks (Morrison et al., 2007). Even the ongoing enhancement of the von Neumann computer architecture is not likely to reduce simulation runtime significantly, as both single- and multi-core scaling face their limits in terms of transistor size (Thompson and Parthasarathy, 2006), energy consumption (Esmaeilzadeh et al., 2011), or communication (Perrin, 2011).

Neuromorphic hardware systems are an alternative to von Neumann computers that alleviates these limitations. Their underlying VLSI microcircuits are especially designed to solve neuron dynamics and can be highly accelerated compared to biological time (Indiveri et al., 2011). For most neuron models whose dynamics can be analytically stated, the evaluation of its equations can be determined either digitally (Plana et al., 2007) by means of numerical methods or with analog circuits that solve the neuron equations intrinsically (Millner et al., 2010). The analog approach has the advantage of maximal parallelism, as all neuron circuits are evolving simultaneously in continuous time. Furthermore, high acceleration factors compared to biological time (e.g. up to 10^5^ reported by Millner et al., 2010), can be achieved by reducing the size of the analog neuron circuits. Nevertheless, many neuromorphic hardware systems are developed for operation in real-time to be applied in sensor applications or medical implants (Fromherz, 2002; Vogels et al., 2005; Levi et al., 2008).

**Figure 1:**
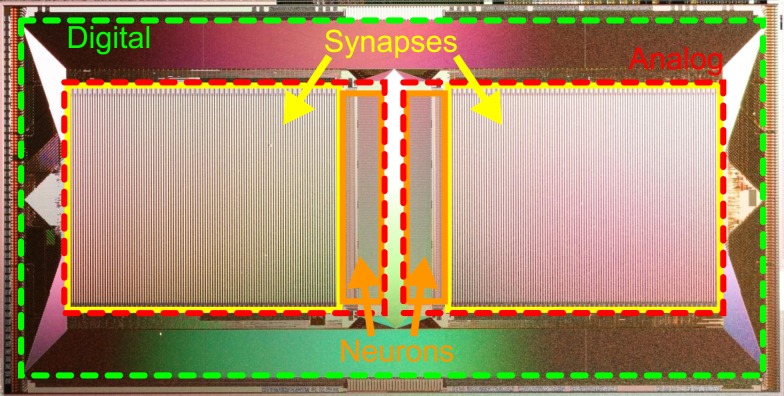
**Photograph of the HICANN (High Input Count Analog Neural Network) chip, the basic building block of the FACETS wafer-scale hardware system**. Notice the large area occupied by mixed-signal synapse circuits (yellow boxes) compared to neuron circuits (orange boxes). A digital communication infrastructure (area between red and green boxes) ensures a high density of connections between neurons on the same and to other HICANN chips.

Typically, the large number of programmable and possibly plastic synapses accounts for the major part of chip resources in neuromorphic hardware systems (Figure 1). Hence, the limited chip area requires a trade-off between the number and size of neurons and their synapses, while providing sufficiently complex dynamics. For example, decreasing the resolution of synaptic weights offers an opportunity to reduce the area required for synapses and therefore allows more synapses on a chip, rendering the synaptic weights discretized.

In this study, we will analyze the consequences of such a weight discretization and propose generic configuration strategies for spike-timing dependent plasticity on discrete weights. Deviations from original models caused by this discretization are quantified by particular benchmarks. In addition, we will investigate further hardware restrictions specific for the *FACETS*^1^ *wafer-scale hardware system* (FACETS, 2010), a pioneering neuromorphic device that implements a large amount of both configurable and plastic synapses (Schemmel et al., 2008, 2010; Brüderle et al., 2011). To this end, custom hardware-inspired synapse models are integrated into a network benchmark using the simulation tool NEST (Gewaltig and Diesmann, 2007). The objective is to determine the smallest hardware implementation of synapses without distorting the behavior of theoretical network models that have been approved by computer simulations.

## 2 Materials and Methods

### 2.1 Spike-timing dependent plasticity

Here, Spike-Timing Dependent Plasticity (STDP) is treated as a pair-based update rule as reviewed by e.g. Morrison et al. (2008). Most pair-based STDP models (Song et al., 2000; van Rossum et al., 2000; Gütig et al., 2003; Morrison et al., 2007) separate weight modifications δ*w* into a spike-timing dependent factor *x*(Δ*t*) and a weight-dependent factor *F*(*w*):

(1) δw(w,Δt)=F(w)x(Δt),

where Δ*t* = *t*_i_ − *t*_j_ denotes the interval between spike times *t_j_* and *t_i_* at the pre- and postsynaptic terminal, respectively. Typically, *x*(Δ*t*) is chosen to be exponentially decaying (e.g. Gerstner et al., 1996; Kempter et al., 1999).

In contrast, the weight-dependence *F*(*w*), which is divided into *F*_+_(*w*) for a causal and *F*_−_(*w*) for an anti-causal spike-timing-dependence, differs between different STDP models. Examples are given in Table 1. As *F*_+_(*w*) is positive and *F*_−_(*w*) negative for all these STDP models, causal relationships (Δ*t* > 0) between pre- and postsynaptic spikes potentiate and anti-causal relationships (Δ*t* < 0) depress synaptic weights.

**Table 1:** **Weight- and spike-timing-dependence of pair-based STDP models: additive, multiplicative, Gütig, van Rossum, and power law model**.

Model name	*F*_+_(*w*)	*F*_−_(*w*)	*x*(Δ*t*)
Additive (Song et al., 2000)	λ	−λα	
Multiplicative (Turrigiano et al., 1998)	λ(1 − *w*)	−λα*w*	
Gütig (Gütig et al., 2003)	λ(1 − *w*)^μ^	−λα*w*^μ^	$exp\left(-\frac{\left|\Delta t\right|}{\tau_{\text{STDP}}}\right)$
van Rossum (van Rossum et al., 2000)	*c*_p_	−*c*_d_*w*	
Power law (Morrison et al., 2007)	λ*w*^μ^	−λα*w*	

**F*_+_ in case of a causal spike-timing-dependence (Δ*t* > 0) and *F_−_* in the anti-causal case (Δ*t* < 0). Throughout this study, the model proposed by Gütig et al. is applied with parameters α = 1.05, λ = 0.005, μ = 0.04, and τ_STDP_ **=** 20 ms in accordance with Song et al. (2000), van Rossum et al. (2000), Rubin et al. (2001), Gütig et al. (2003), Morrison et al. (2008)*.

In this study, the *intermediate Gütig STDP model* (bounded to the weight range [0, 1]) is chosen as an example STDP model. It represents a mixture of the multiplicative (μ = 1) and additive (μ = 0) STDP model and has been shown to provide stability in competitive synaptic learning (Gütig et al., 2003). Nevertheless, the following studies can be applied to any pair-based STDP model with exponentially decaying time-dependence, e.g. all models listed in Table 1.

### 2.2 Synapses in large-scale hardware systems

**Figure 2:**
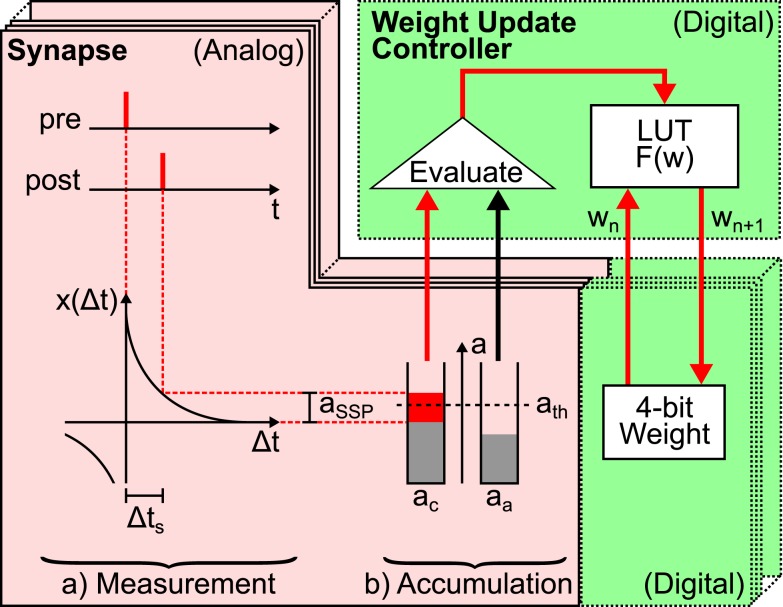
**Schematic drawing of local hardware synapses which are consecutively processed by a global weight update controller**. Analog circuits are highlighted in red (with solid frame) and digital circuits in green (dashed frames). The spike-timing-dependence (here one standard spike pair (SSP) with Δ*t*_s_, see text) between the pre- and postsynaptic neuron is (a) measured (here *a*_SSP_) and (b) accumulated (here to *a*_c_ in case of a causal spike pair, *a*_a_ for anti-causal spike pairs is not affected). Then, the global weight update controller evaluates the accumulated spike-timing-dependence by means of a crossed threshold *a*_th_ (here *a*_c_ > *a*_th_) and modifies the digital weight of the hardware synapse accordingly. The new synaptic weight *w_n + 1_* is retrieved from the LUT according to the accumulated spike-timing-dependence and the current weight *w_n_* and is written back to the hardware synapse. The analog measurement and accumulation circuit is furthermore minimized by using the reduced symmetric nearest-neighbor spike pairing scheme (Morrison et al., 2008): instead of considering all past and future spikes (all-to-all spike pairing scheme), only the latest and the following spike at both terminals of the synapse are taken into account.

The FACETS wafer-scale hardware system (Schemmel et al., 2008, 2010; Brüderle et al., 2011) represents an example for a possible synapse size reduction in neuromorphic hardware systems. Figure 2 schematizes the hardware implementation of a synapse enabling STDP similar as presented in Schemmel et al. (2006) and Schemmel et al. (2007). It provides the functionality to store the value of the synaptic weight, to measure the spike-timing-dependence between pre- and postsynaptic spikes and to update the synaptic weight according to this measurement. Synapse density is maximized by separating the *accumulation* of the spike-timing-dependence *x*(Δ*t*) and the *weight update controller*, which is the hardware implementation of *F*(*w*). This allows 4·10^7^ synapses on a single wafer (Schemmel et al., 2010).

Synaptic dynamics in the FACETS wafer-scale hardware system exploits the fact that weight dynamics typically evolves slower than electrical neuronal activity (Morrison et al., 2007; Kunkel et al., 2011). Therefore, weight updates can be divided into two steps (Figure 2). First, a measuring and accumulation step which locally determines the relative spike times between pairs of neurons and thus *x*(Δ*t*). This stage is designed in analog hardware (red area in Figure 2), as analog measurement and accumulation circuits require less chip resources compared to digital realizations thereof. Second, the digital weight update controller (upper green area in Figure 2) implements *F*(*w*) based on the previous analog result. A global weight update controller^2^ is responsible for the consecutive updates of many synapses (Schemmel et al., 2006) and hence limits the maximal rate at which a synapse can be updated, the update controller frequency *v_c_*.

Sharing one weight update controller reduces synapses to small analog measurement and accumulation circuits as well as a digital circuit that implements the synaptic weight (Figure 2). The area required to implement these digital weights with a resolution of *r* bits is proportional to 2*^r^*, the number of discrete weights. Consequently, assuming the analog circuits to be fixed in size, the size of a synapse is determined by its weight storage exponentially growing with the weight resolution. E.g. the FACETS wafer-scale hardware system has a weight resolution of *r* = 4 bits, letting the previously described circuits (analog and digital) equally sized on the chip.

Modifications in the layout of synapse circuits are time-consuming and involve expensive re-manufacturing of chips. Thus, the configuration of connections between neurons is designed flexible enough to avoid these modifications and provide a general-purpose modeling environment (Schemmel et al., 2010). For the same reason, STDP is conform to the majority of available update rules. The STDP models listed in Table 1 share the same time-dependence *x*(Δ*t*). Its exponential shape is mimicked by small analog circuit not allowing for other time-dependencies (Schemmel et al., 2006, 2007). The widely differing weight-dependences *F*(*w*), on the other hand, are programmable into the weight update controller. Due to limited weight update controller resources, arithmetic operations *F*(*w*) as listed in Table 1 are not realizable and are replaced by a programmable look-up table (LUT; Schemmel et al., 2006).

Such a LUT lists, for each discrete weight, the resulting weights in case of causal or anti-causal spike-timing-dependence between pre- and postsynaptic spikes. Instead of performing arithmetic operations during each weight update (equation 1), LUTs are used as a recallable memory consisting of precalculated weight modifications. Hence, LUTs do not limit the flexibility of weight updates if their weight-dependence (Table 1) does not change over time. Throughout this study, we prefer the concept of LUTs to arithmetic operations, because we like to focus on the discretized weight space, a state space of limited dimension.

In addition to STDP, the FACETS wafer-scale hardware system also supports a variant of short-term plasticity mechanisms according to (Tsodyks and Markram, 1997; Bi and Poo, 1998; Schemmel et al., 2007), which however leaves synaptic weights unchanged and therefore lies outside the scope of this study.

### 2.3 Discretization of synaptic weights

Continuous weight values *w_c_ *∈ [0, 1], as assumed for the STDP models listed in Table 1, are transformed into *r*-bit coded discrete weight values *w*_d_:

(2) $$w_{\text{d}}=c\left\lfloor \frac{w_{\text{c}}}{c}+\frac{1}{2}\right\rfloor \;\text{for}\;w_{\text{c}}\in I$$

where *c* = 1/(2*^r^* − 1) denotes the width of a bin and ⌊x⌋ the floor-function, the largest integer less than or equal to *x*. This procedure divides the range of weight values *I* = [0, 1] into 2*^r^* bins. The term 1/2 allows for a correct discretization of weight values near the borders of *I*, effectively dividing the width of the ending bins (otherwise, only *w*_c_ = 1 would be mapped to *w*_d_ = 1).

### 2.4 Discretization of spike-timing dependent plasticity

A single weight update, resulting from a pre- and postsynaptic spike, might be too fine grained to be captured by a low weight resolution (equation 2). Therefore, it is necessary to accumulate the effect of weight updates of several consecutive spike pairs in order to reach the next discrete weight value (equation 2; Figure 2). This is equivalent to state that the implementation of the STDP model assumes additive features for ms range intervals. To this end, we define a *standard spike pair* (SSP) as a spike pair with a time interval between a pre- and postsynaptic spike of Δ*t*_s_ = 10 ms in accordance to biological measurements by Bi and Poo (2001), Sjöström et al. (2001), Markram (2006) in order to provide a standardized measure for the spike-timing-dependence. This time interval is chosen arbitrarily defining the granularity only (fine enough for the weight resolutions of interest) and is valid for both pre-post and post-pre spike pairs, as *x*(Δ*t*) takes its absolute value.

The values for a LUT are constructed as follows. First, the parameters *r* (weight resolution) and *n* (number of SSPs consecutively applied for an accumulated weight update) as well as the STDP rule-specific parameters τ_STDP_, λ, μ, α (Table 1) are chosen. Next, starting with a discrete weight *w*_d_, weight updates *δw*(*w*, Δ*t*_s_) specified by equation (1) are recursively applied *n* times in continuous weight space using either exclusively *F*_+_(*w*) or *F*_−_(*w*). This results in two accumulated weight updates Δ*w*_+/−_, one for each weight-dependence *F*_+/−_(*w*). Finally, the resulting weight value in continuous space is according to equation (2) transformed back to its discrete representation. This process is then carried out for each possible discrete weight value *w*_d_ (Table 2). We will further compare different LUTs letting *n* be a free parameter. In the following a *weight update* refers to Δ*w*, if not specified otherwise.

**Table 2:** **Example look-up table for a weight resolution of *r* = 2 bits and *n* = 100 SSPs**.

*w*_d_	*w*_+_	*w*_−_
0	$\frac{1}{3}$	0
$\frac{1}{3}$	$\frac{2}{3}$	0
$\frac{2}{3}$	1	$\frac{1}{3}$
1	1	$\frac{2}{3}$

*Discrete weight *w*_d_ and the resulting weight increments *w*_+/−_ = *w*_d_ + Δ*w*_+/−_ for causal and anti-causal weight-dependences*.

Although we are focusing on the Gütig STDP model, the updated weight values can in general under- or over-run the allowed weight interval *I* due to finite weight updates Δ*w*. In this case, the weight is clipped to its minimum or maximum value, respectively.

### 2.5 Equilibrium weight distributions

We analyze long-term effects of weight discretization by studying the equilibrium weight distribution of a synapse that is subject to Poissonian pre- and postsynaptic firing. Thus, potentiation and depression are equally probable (*p*_d_ = *p*_p_ = 1/2). Equilibrium weight distributions in discrete weight space of low resolution (between 2 and 10 bits) are compared to those with high resolution (16 bits) via the mean squared error *MSE*_eq_. Consecutive weight updates are performed based on precalculated LUTs.

Equilibrium weight distributions of discrete weights for a given weight resolution of *r* bits are calculated as follows. First, a LUT for 2*^r^* discrete weights is configured with *n* SSPs. Initially, all 2*^r^* discrete weight values *w_i_* have the same probability *P*_i,0_ = 1/2*^r^*. For a compact description, the discrete weights *w_i_* are mapped to a 2*^r^* dimensional space with unit vectors ${\vec{e}}_{\text{i}}\in {\mathbb{N}}^{2^{r}}$. Then, for each iteration cycle *j*, the probability distribution is defined by ${\vec{P}}_{\text{j}}=\sum_{\text{i}=0}^{2^{r}-1}P_{\text{i},\text{j}-1}\left(p_{\text{P}}{\vec{e}}_{\text{c}}+p_{\text{d}}{\vec{e}}_{\text{a}}\right)$ where *P_i,j−1_* is the probability for each discrete weight value *w*_i_ of the previous iteration cycle *j* − 1. The indices of ${\vec{e}}_{\text{c}}$ and ${\vec{e}}_{\text{a}}$ are those of the resulting discrete weight values *w*_i_ in case of a causal and anti-causal weight update, respectively, and are represented by the LUT. We define an equilibrium state as reached if the Euclidean norm $\|{\vec{P}}_{\text{j}-1}-{\vec{P}}_{\text{j}}\|$ is smaller than a threshold *h* = 10^−12^.

An analytical approach for obtaining equilibrium weight distributions is derived in Section 6.1.

### 2.6 Spiking network benchmarks

In addition to the behavior under Poissonian noise, we study the impact of discretized weights with a software implementation of hardware synapses, enabling us to analyze synapses in isolation as well as in network benchmarks. The design of our simulation environment is flexible enough to take further hardware constraints and biological applications into account.

#### 2.6.1 Software implementation of hardware synapses

The hardware constraints considered in this study are implemented as a customized synapse model within the framework of the NEST simulation tool (Gewaltig and Diesmann, 2007), allowing their well controlled application in simulator-based studies on large-scale neural networks. The basic properties of such a *hardware-inspired synapse* *model* are described as follows and are illustrated in Figures 2 and 5.

For each LUT configuration defined by its weight resolution *r* and number *n* of SSPs, the threshold for allowing weight updates is set to

(3) $$a_{\text{th}}=n\cdot a_{\text{SSP}},$$

defining *a* = ∑*_i_x*(Δ*t_i_*) as the *spike pair accumulation* for arbitrary intervals. Here, a single SSP is used, setting *a* = *a*_SSP_ = *x*(Δ*t*_s_). If either the causal or anti-causal spike pair accumulation *a*_c/a_ crosses the threshold *a*_th_, the synapse is “tagged” for a weight update. At the next cycle of the weight update controller all tagged synapses are updated according to the LUT. Afterward, the spike pair accumulation (causal or anti-causal) is reset to zero. Untagged synapses remain unprocessed by the update controller, and spike pairs are further accumulated without performing any weight update. If a synapse accumulates *a*_c_ and *a*_a_ above threshold between two cycles of the weight update controller, both are reset to zero without updating the synaptic weight.

This threshold process implies that the frequency *v*_w_ of weight updates is dependent on *n*, which in turn determines the threshold *a*_th_, but also on the firing rates and the correlation between the pre- and postsynaptic spike train. In general, *a* increases faster with higher firing rates or higher correlations. To circumvent these dependencies on network dynamics, we will use *n* as a generalized description for the weight update frequency *v*_w_. The weight update frequency *v*_w_ should not be confused with the update controller frequency *v*_c_, with which is checked for threshold crossings and hence limits *v*_w_.

Furthermore, we have implemented a *reference synapse model* in NEST, which is based on Gütig et al. (2003). It has the reduction of employing nearest-neighbor instead of all-to-all spike pairing (Morrison et al., 2008).

All simulations involving synapses are simulated with NEST. Spike trains are applied to built-in *parrot neurons*, that simply repeat their input, in order to control pre- and postsynaptic spike trains to interconnecting synapses.

#### 2.6.2 Single synapse benchmark

We compare the weight evolutions of hardware-inspired and reference synapses receiving correlated pre- and postsynaptic spike trains, drawn from a multiple interaction process (MIP; Kuhn et al., 2003). This process introduces excess synchrony between two realizations by randomly thinning a template Poisson process. SSPs are then obtained by shifting one of the processes by Δ*t*_s_.

In this first scenario the spike pair accumulation *a* is checked for crossing *a*_th_ with a frequency of *v*_c_ = 10 Hz to focus on the effects of discrete weights only. This frequency is equal to the simulation step size, preventing the spike pair accumulation from overshooting the threshold *a*_th_ without eliciting a weight update.

Synaptic weights are recorded in time steps of 3 s for an overall period of 150 s and are averaged over 30 random MIP realizations. Afterward the mean weight at each recorded time step is compared between the hardware-inspired and the reference synapse model by applying the mean squared error *MSE_w_*.

#### 2.6.3 Network benchmarks

The detection of presynaptic synchrony is taken as a benchmark for synapse implementations. Two populations of 10 neurons each converge to an integrate-and-fire neuron with exponentially decaying synaptic conductances (see schematic in Figure 7A and model description in Tables 7 and 8) by either hardware-inspired or reference synapses. These synapses are excitatory, and their initial weights are drawn randomly from a uniform distribution over [0, 1). The amplitude of the postsynaptic conductance is *wg*_max_ with *g*_max_ = 100 nS. One population draws its spikes from a MIP with correlation coefficient *c* (Kuhn et al., 2003), the other from a Poisson process (MIP with c → 0). We choose presynaptic firing rates of 7.2 Hz such that the target neuron settles at a firing rate of 2–22 Hz depending on the synapse model. The exact postsynaptic firing rate is of minor importance as long as the synaptic weights reach an equilibrium state. The synaptic weights are recorded for 2,000 s with a sampling frequency of 0.1 Hz. The two resulting weight distributions are compared applying the Mann–Whitney U test (Mann and Whitney, 1947).

##### 2.6.3.1 Further constraints

Not only the discretization of synaptic weights, but also the update controller frequency *v*_c_ and the reset behavior are constraints of the FACETS wafer-scale hardware system.

To study effects caused by a limited update controller frequency, we choose *v*_c_ such that the interval between sequent cycles is a multiple of the simulator time step. Consequently weight updates can only occur on a time grid.

A *common reset* means that both the causal and anti-causal spike pair accumulations are reset, although only either *a*_c_ or *a*_a_ has crossed *a*_th_. Because the common reset requires only one reset line instead of two, it decreases the chip resources of synapses and is implemented in the current FACETS wafer-scale hardware system.

As a basis for a possible compensation mechanism for the common reset, we suggest analog-to-digital converters (ADCs) with a 4-bit resolution that read out the spike pair accumulations. Such ADCs require only a small chip area in the global weight update controller compared to the large area occupied by additional reset lines covering all synapses and are therefore resource saving alternatives to second reset lines. An ADC allows to compare the spike pair accumulations against multiple thresholds. Implementations of the common reset as well as ADCs are added to the existing software model. For multiple thresholds, the same number of LUTs is needed that have to be chosen carefully. To provide symmetry within the order of consecutive causal and anti-causal weight updates, the spike pair accumulation (causal or anti-causal) that dominates in means of crossing a higher threshold is applied first.

##### 2.6.3.2 Peri-stimulus-time-histograms

The difference between static and STDP synapses on eliciting postsynaptic spikes in the above network benchmark can be analyzed with peri-stimulus-time-histograms (PSTHs). Here, PSTHs show the probability of postsynaptic spike occurrences in dependence on the delay between a presynaptic trigger and its following postsynaptic spike. Spike times are recorded within the last third of an elongated simulation of 3,000 s with *c* = 0.025. During the last 1,000 s the mean weights are already in their equilibrium state, but are still fluctuating around it. The first spike of any two presynaptic spikes within a time window of Δ*t*_on_ = 1 ms is used as a trigger. The length of Δ*t*_on_ is chosen small compared to the membrane time constant τ_m_ = 15 ms, such that the excitatory postsynaptic potentials of both presynaptic spikes overlap each other and increase the probability of eliciting a postsynaptic spike. On the other hand Δ*t*_on_ is chosen large enough to not only include the simultaneous spikes generated by the MIP, but also include coincident spikes within the uncorrelated presynaptic population.

### 2.7 Hardware variations

In contrast to arithmetic operations in software models, analog circuits vary due to the manufacturing process, although they are identically designed. The choice of precision for all building blocks should be governed by those that distort network functionality most. In this study, we assume that variations within the analog measurement and accumulation circuits are likely to be a key requirement for these choices, as they operate on the lowest level of STDP. Circuit variations are measured and compared between the causal and anti-causal part within a synapse and between synapses. All measurements are carried out with the FACETS chip-based hardware system (Schemmel et al., 2006, 2007) with hardware parameters listed in Table 6. The FACETS chip-based hardware system shares a conceptually nearly identical STDP circuit with the FACETS wafer-scale hardware system (for details see Section 2) which was still in the assembly process at the course of this study. The hardware measurements are written in PyNN (Davison and Frégnac, 2006) and use the workflow described in (Brüderle et al., 2011).

#### 2.7.1 Measurement

The circuit variations due to production imperfection are measured by recording *STDP curves* and comparing their integrals for Δ*t* > 0 and Δ*t* < 0. The curves are recorded by applying equidistant pairs of pre- and postsynaptic spikes with a predefined latency Δ*t*. Presynaptic spikes can be fed into the hardware precisely. However, in contrast to NEST’s parrot neurons, postsynaptic spikes are not directly adjustable and therefore has to be evoked by several synchronous external triggers (for details see Section 6.3). After discarding the first 10 spike pairs to ensure regular firing, the pre- and postsynaptic spike trains are shifted until the desired latency Δ*t* is measured. Due to the low spike pair frequency of 10 Hz, only the correlations within and not between the spike pairs are accumulated. The number *N* of consecutive spike pairs is increased until the threshold is crossed and hence a correlation flag is set (Figure 8A). The inverse of this number versus Δ*t* is called an STDP curve. Such curves were recorded for 252 synapses within one synapse column, the remaining 4 synapses in this column were discarded.

For each STDP curve the total area *A*_t_ = *A*_a_ + *A*_c_ is calculated and normalized by the mean $\bar{A_{\text{abs}}}$ of the absolute area *A*_abs_ = |*A*_a_| + |*A*_c_| over all STDP curves. Ideally, *A*_t_ would vanish if both circuits are manufactured identically. The standard deviation σ_a_ (assuming Gaussian distributed measurement data) of these normalized total areas *A*_t_ is taken as one measure for circuit variations. Besides this asymmetry which measures the variation *within* a synapse, a measure for variation *across* synapses is the standard deviation σ_t_ of the absolute areas *A*_abs_. Therefore the absolute areas *A*_abs_ under each STDP curve are again normalized by $\bar{A_{\text{abs}}}$ and furthermore the mean of all these normalized absolute areas is subtracted.

#### 2.7.2 Software analysis

In order to predict the effects of the previously measured variations on the network benchmark, these variations are integrated into computer simulations. The thresholds for the causal and anti-causal spike pair accumulations are drawn from two overlaying Gaussian distributions defined by the ideal thresholds (equation 3) and their variations σ_t_, σ_a_. Again, the same network benchmark as described above is used, but with a fixed correlation coefficient of *c* = 0.025 and an 8-bit LUT configured with *n* = 12 SSPs.

## 3 Results

Synaptic weights of the FACETS wafer-scale hardware system (Schemmel et al., 2010) have a 4-bit resolution. We show that such a weight resolution is enough to exhibit learning in a neural network benchmark for synchrony detection. To this end, we analyze the effects of weight discretization in three steps as summarized in Table 3.

**Table 3:** **Outline of analyses on the effects of weight discretization and further hardware constraints**.

Description	Results	Methods
**LOOK-UP TABLE ANALYSIS**		
Basic analyses on the configuration of STDP on discrete weights by means of look-up tables (A)	A) Section 3.1	A) Sections 2.3 and 2.4
and their long-term dynamics (B).	B) Section 3.2	B) Section 2.5
**SPIKING NETWORK BENCHMARKS**		
Software implementation of hardware-inspired synapses with discrete weights for application in spiking neural environments (C).		C) Section 2.6.1
Analyses of their effects on short-term weight dynamics in single synapses (D)	D) Section 3.3.1	D) Section 2.6.2
and neural networks (E).	E) Section 3.3.2	E) Section 2.6.3
Analyses on how additional hardware constraints effect the network benchmark (F).	F) Section 3.3.3	F) Section 2.6.3
**HARDWARE MEASUREMENTS**		
Measurement of hardware variations (G)	G) Section 3.4	G) Section 2.7.1
and computer simulations analyzing their effects on the network benchmark (H).	H) Section 3.4	H) Section 2.7.2

### 3.1 Dynamic range of STDP on discrete weights

We choose the configuration of STDP on discrete weights according to Sections 2.3 and 2.4 to obtain weight dynamics comparable to that in continuous weight space. Each configuration can be described by a LUT “projecting” each discrete weight to new values, one for potentiation and one for depression (Table 4). For a given weight resolution *r* the free configuration parameter *n* (number of SSPs) has to be adjusted to avoid a further reduction of the usable weight resolution by *dead discrete weights*. Dead discrete weights are defined as weights projecting to themselves in case of both potentiation and depression or not receiving any projections from other discrete weights. The percentage of dead discrete weights *d* defines the lower and upper limit of feasible values for *n*, the *dynamic range*. The absolute value of the interval within a SSP (Δ*t*_s_) is an arbitrary choice merely defining the granularity, but does not affect the results (not shown). Note that spike-timing precision *in vivo*, which is observed for high dimensional input such as dense noise and natural scenes, goes rarely beyond 5–10 ms (Butts et al., 2007; Desbordes et al., 2008, 2010; Marre et al., 2009; J. Frégnac, personal communication), and the choice of 10 ms as a granular step is thus justified biologically.

**Table 4:** **Look-up tables for different numbers *n* of SSPs**.

*w_d_*	*w_+_*	*w_−_*	*w_d_*	*w_+_*	*w_−_*	*w_d_*	*w_+_*	*w_−_*
0	$\frac{1}{3}$	0	0	$\frac{1}{3}$	0	0	$\frac{2}{3}$	0
$\frac{1}{3}$	$\frac{2}{3}$	0	$\frac{1}{3}$	$\frac{1}{3}$	$\frac{1}{3}$	$\frac{1}{3}$	1	0
$\frac{2}{3}$	1	$\frac{1}{3}$	$\frac{2}{3}$	$\frac{2}{3}$	$\frac{2}{3}$	$\frac{2}{3}$	1	0
1	1	$\frac{2}{3}$	1	1	$\frac{2}{3}$	1	1	0
(a)			(b)			(c)		

*(a) As in Table 2 (*n* = 100), which results in a LUT as expected. Weights are either potentiated or depressed through the entire table. (b) *n* = 60, which is too low, because the discrete weights $\frac{1}{3}$ and $\frac{2}{3}$ are projecting exclusively to themselves. (c) *n* = 350, which is too large, because for *w_+_* the discrete weight 0 is mapped right to $\frac{2}{3}$ (and for *w*_-_ the weight 1 is mapped to 0), thus $\frac{1}{3}$ is never reached*.

**Figure 3:**
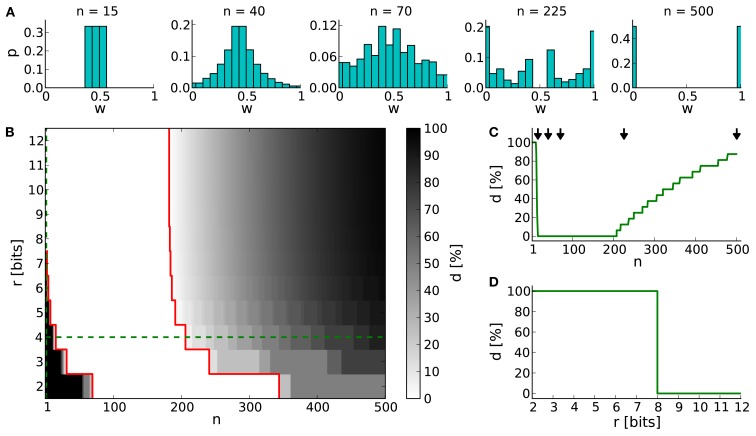
**The dynamic range for configurations of STDP on discrete weights**. **(A)** Equilibrium weight distributions for a 4-bit weight resolution: Intermediate discrete weights partly project to themselves (*n* = 15). The equilibrium weight distribution widens with an increasing number of SSPs (*n* = 40 and 70). For a large number of SSPs (*n* = 225 and 500) the intermediate discrete weights do not receive projections from others. **(B)** Percentage of dead discrete weights *d*. The limits of the dynamic range (*d* = 0%) are highlighted in red. The limit toward low numbers of SSPs (*n* = 15 in case of *r* = 4 bits) is caused by rounding effects (equation 2), whereas the upper limit (*n* = 206 in case of *r* = 4 bits) is caused by too large weight updates. Green dashed lines indicate cross sections shown in **(C,D)**. **(C)** Cross section of **(B)** at a 4-bit weight resolution. The histograms shown in **(A)** are depicted with arrows. **(D)** Cross section of **(B)** at *n* = 1.

Generally, low values of *n* realize frequent, small weight updates. However, if *n* is too low, some discrete weights may project to themselves (see rounding in equation 2) and prevent synaptic weights from evolving dynamically (see Table 4; *n* = 15 in Figure 3A).

On the other hand, if *n* exceeds the upper limit of the dynamic range, intermediate discrete weights may not be reached by others. Rare, large weight updates favor projections to discrete weights near the borders of the weight range *I* and lead to a bimodal equilibrium weight distribution as shown in Table 4 and Figure 3A (*n* = 500).

The lower limit of the dynamic range decreases with increasing resolution (Figure 3B). Compared to a 4-bit weight resolution, an 8-bit weight resolution is sufficiently high to resolve weight updates down to a single SSP (Figure 3D). This allows frequent weight updates comparable to weight evolutions in continuous weight space. The upper limit of the dynamic range does not change over increasing weight resolutions, but is critical for limited update controller frequencies as investigated in Section 3.3.

### 3.2 Equilibrium weight distributions

**Figure 4:**
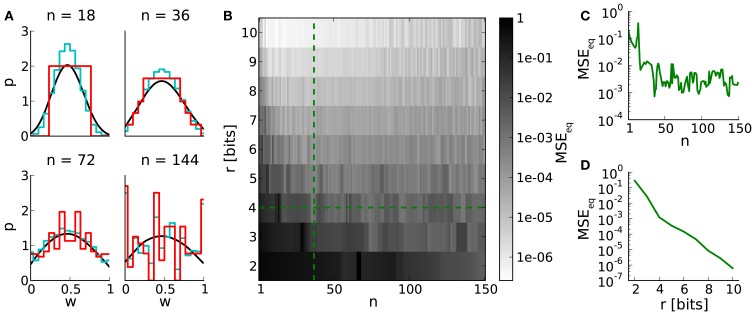
**Equilibrium weight distributions (long-term weight evolutions) for configurations of STDP on discrete weights**. **(A)** Equilibrium weight distributions for weight resolutions of *r* = 4 bits (red) and *r* = 16 bits (cyan). Both distributions are displayed in 4-bit sampling, for better comparison. Black curves depict the analytical approach. We have chosen *j* = 10^5^ iterations for generating each discrete weight distribution to ensure convergence to the equilibrium state. **(B)** Mean squared error *MSE*_eq_ between the equilibrium weight distributions for weight resolutions *r* and the reference weight resolution of 16 bits versus the number *n* of SSPs. **(C,D)** Cross sections of **(B)** at *r* = 4 bits and *n* = 36, respectively.

Studying learning in neural networks may span long periods of time. Therefore we analyze equilibrium weight distributions being the temporal limit of Poissonian distributed pre- and postsynaptic spiking. These distributions are obtained by applying random walks on LUTs with uniformly distributed occurrences of potentiations and depressions (Section 2.5). Figure 4A shows i.a. boundary effects caused by LUTs configured within the upper part of the dynamic range. E.g. for *n* = 144, the relative frequencies of both boundary values are increased due to large weight steps (red and cyan distributions). Frequent weights, in turn, increase the probability of weights to which they project (according to the LUT). This effect decreases with the number of look-ups, due to the random nature of the stimulus, however, causing intermediate weight values to occur at higher probability.

The impact of weight discretization on long-term weight dynamics is quantified by comparing equilibrium weight distributions between low and high weight resolutions. Weight discretization involves distortions caused by rounding effects for small *n* (equation 2; Figure 3) and boundary effects for high *n* (Figures 4A,C). High weight resolutions can compensate for rounding effects, but not for boundary effects (Figure 4B).

This analysis on long-term weight dynamics (Figure 4C) refines the choice for *n* roughly estimated by the dynamic range (Figure 3C).

### 3.3 Spiking network benchmarks

**Figure 5:**
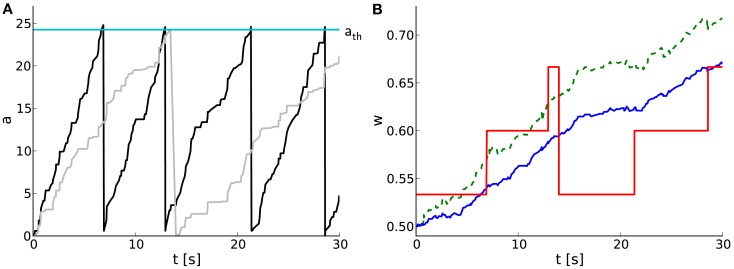
**Software implementation of STDP on discrete weights in spiking neural networks**. **(A)** Temporal evolution of spike pair accumulations *a* (dimensionless) for causal (black) and anti-causal (gray) spike-timing-dependences. If *a* crosses the threshold *a*_th_ (cyan), the weight is updated and *a* is reset to zero. Pre- and postsynaptic spike trains are generated by a MIP with *c* = 0.5 and *r* = 10 Hz. **(B)** Corresponding weight evolution (solid red) for a 4-bit weight resolution and a LUT configured with *n* = 30. The weight evolution of the reference synapse model with continuous weights, but a reduced symmetric nearest-neighbor spike pairing scheme is depicted in solid blue. It differs from that of a synapse model with continuous weights and an all-to-all spike pairing scheme (dashed green).

We extend the above studies on temporal limits by analyses on short-term dynamics with unequal probabilities for potentiation *p*_p_ and depression *p*_d_. A hardware-inspired synapse model is used in computer simulations of spiking neural networks, of which an example of typical dynamics is shown in Figure 5. As the pre- and postsynaptic spike trains are correlated in a causal fashion, the causal spike pair accumulation increases faster than the anti-causal one (Figure 5A). It crosses the threshold twice, evoking two potentiation steps (at around 7 and 13 s) before the anti-causal spike pair accumulation evokes a depression at around 14 s (Figures 5A,B). The first two potentiations project to the subsequent entry of the LUT, whereas the following depression rounds to the next but one discrete weight (omitting one entry in the LUT) due to the asymmetry measure α in the STDP model by Gütig et al. (2003).

#### 3.3.1 Single synapse benchmark

**Figure 6:**
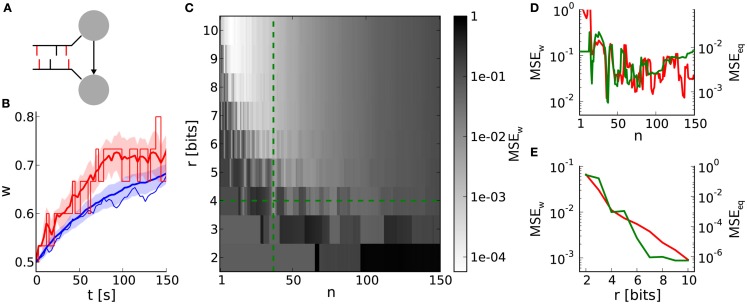
**Weight evolution of a single synapse with discrete weights**. **(A)** Network layout for single synapse analyses. An STDP synapse (arrow) connects two neurons receiving correlated spike trains with correlation coefficient *c* (correlated spikes in red bars). **(B)** Example weight traces for the hardware-inspired (*r* = 4 bits, *n* = 36 in red) and reference synapse model (blue). Means and standard deviations over 30 realizations are plotted as bold lines and shaded areas, respectively. The single weight traces for one arbitrarily chosen random seed are depicted as thin lines. We applied a correlation coefficient *c* = 0.2, an initial weight *w*_0_ = 0.5 and firing rates of 10 Hz. The results persist qualitatively for differing values staying within biologically relevant ranges (not shown here). **(C)** Mean squared error *MSE_w_* between the mean weight traces as shown in **(A)** over the weight resolution *r* and the number *n* of SSPs. The parameters *c*, *w*_0_, and the firing rates are chosen as in **(B)**. Other values for *c* and *w*_0_ do not change the results qualitatively. **(D,E)** Cross sections of **(C)** at *r* = 4 bits and *n* = 36 in green. Red curves are adapted from Figures 4C,D.

This benchmark compares single weight traces between hardware-inspired and reference synapses (Section 2.6.2). A synapse receives correlated pre- and postsynaptic input (Figure 6A) resulting in weight dynamics as shown in Figure 6B. The standard deviation for discrete weights (hardware-inspired synapse model) is larger than that for continuous weights (reference model). This difference is caused by rare, large weight jumps (induced by high *n*) also responsible for the broadening of equilibrium weight distributions (Figure 4A). Consequently, the standard deviation increases further with decreasing weight resolutions (not shown here).

The dependence of the deviation between discrete and continuous weight traces on the weight resolution *r* and the number *n* of SSPs is qualitatively comparable to that of comparisons between equilibrium weight distributions (Figures 6D,E). This similarity, especially in dependence on *n* (Figure 6D), emphasizes the crucial impact of LUT configurations on both short- and long-term weight dynamics.

To further illustrate underlying rounding effects when configuring LUTs, the asymmetry value α in Gütig’s STDP model can be taken as an example. In an extreme case both potentiation and depression are rounded down (compare weight step size for potentiation and depression in Figure 5B). This would increase the originally slight asymmetry drastically and therefore enlarge the distortion caused by weight discretization.

The weight update frequency *v*_w_ is determined by the weight resolution *r* and the number *n* of SSPs. High frequencies are beneficial for chronologically keeping up with weight evolutions in continuous weight space. They can be realized by small numbers of SSPs lowering the threshold *a*_th_ (equation 3). On the other hand, rounding effects in the LUT configuration deteriorate for too small numbers of SSPs (Figure 6D). In case of a weight resolution *r* = 4 bits (*r* = 8 bits) choosing *n* = 36 (*n* = 12) for the LUT configuration represents a good balance between a high weight update frequency and proper both short- and long-term weight dynamics (Figures 3B, 4B and 6C). Note that *n* can be chosen smaller for higher weight resolutions, because the distorting impact of rounding effects decreases.

#### 3.3.2 Network benchmark: synchrony detection

**Figure 7:**
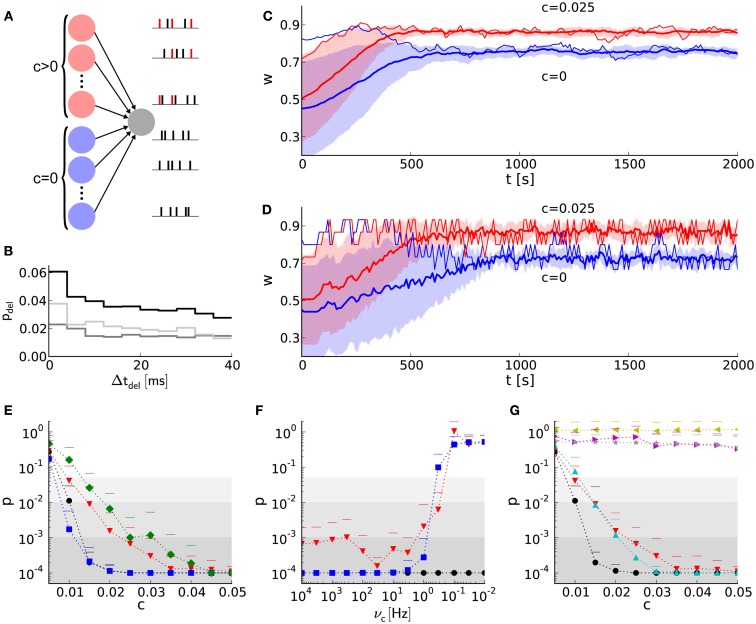
**Learning with discrete weights in a neural network benchmark for synchrony detection**. **(A)** Layout of the network benchmark. Two populations of presynaptic neurons are connected to a postsynaptic neuron. On the right, example spike trains of the presynaptic neurons are shown. Red spikes indicate correlated firing due to shared spikes. **(B)** PSTH for static synapses and STDP reference synapses. The light gray histogram shows the difference between a simulation with STDP reference synapses (black) and static synapses (dark gray). **(C)** The mean weight traces (thick lines) and their standard deviations (shaded areas) for both populations of afferent synapses using the reference synapses model. Thin lines represent single synapses randomly chosen for each population. **(D)** As in **(B)**, but with the hardware-inspired synapse model (*r* = 4 bits and *n* = 36). **(E)** The probability (*p*-value of Mann–Whitney U test) of having the same median of weights within both groups of synapses (with correlated and correlated input) at *t* = 2,000 s versus the correlation coefficient *c*. The hardware-inspired synapses model is represented in red (*r* = 4 bits and *n* = 36), green (*r* = 4 bits and *n* = 36) and blue (*r* = 8 bits and *n* = 12). Black depicts the reference synapse model (*r* = 64 bits). The background shading represents the significance levels: *p* < 0.05, *p* < 0.01, and *p* < 0.001. **(F)** Dependence of the *p*-value on the update controller frequency *v*_c_ for *c* = 0.025. Colors as in **(E)**. **(G)** Black and red trace as in **(E)**. Additionally, *p*-values for hardware-inspired synapses with common resets are plotted in yellow (*r* = 4 bits and *n* = 36) and magenta (*r* = 8 bits and *n* = 12). Compensations with ADCs are depicted in gray (*r* = 4 bits and *n* = 15–45 in steps of 2) and cyan (*r* = 8 bits and *n* = 1–46 in steps of 3).

Not only exact weight traces of single synapses (Section 3.3), but rather those of synapse populations are crucial to fulfill tasks, e.g. the detection of synchronous firing within neural networks. The principle of synchrony detection is a crucial feature of various neural networks with plasticity, e.g. reported by Senn et al. (1998), Kuba et al. (2002), Davison et al. (2009), El Boustani et al. (2012). Here, it is introduced by means of an elementary benchmark neural network (Figure 7A; Section 3), using the hardware-inspired or reference synapse model, respectively.

Figure 7B shows a delay distribution of postsynaptic spike occurrences, relative to the trigger onset, synchronous presynaptic firing (Section 2). For the shown range of Δ*t*_del_, the postsynaptic neuron is more likely to fire if connected with static (dark gray trace) instead of STDP (black trace) synapses. The correlated population causes its afferent synapses to strengthen more compared to those from the uncorrelated population. This can be seen in Figure 7C, where *w* saturates at different values (*t* ≈ 700 s). The same effect can be observed for discretized weights in Figure 7D. For Δ*t*_del_ > 170 ms the delay distribution for static synapses is larger than that for STDP synapses (not shown here), because such delayed postsynaptic spikes are barely influenced by their presynaptic counterparts. This is due to small time constants of the postsynaptic neuron (see τ_m_ = *C*_m_/*g*_L_ and τ_syn_ in Tables 7 and 8) compared to Δ*t*_del_.

Figure 7E shows the *p*-values of the Mann–Whitney U test applied to both groups of synaptic weights at *t* = 2,000 s for different configurations of weight resolution *r* and number *n* of SSPs. Generally, *p*-values (probability of having the same median within both groups of weights) decrease with an increasing correlation coefficient. Although applying previously selected “healthy” LUT configurations, weight discretization changes the required correlation coefficient for reaching significance level (gray shaded areas). Incrementing the weight resolution while retaining the number of SSPs *n* does not change the *p*-values significantly. Low weight resolutions cause larger spacings between discrete weights that can further facilitate the distinction between both medians (for *n* = 36 compare *r* = 4 bits to *r* = 8 bits in Figure 7E). However, reducing *n* for high weight resolutions shortens the accumulation period and consequently allows the synapses to capture fluctuations in *a* on smaller time scales. This improves the *p*-value, but is inconvenient for low weight resolutions, because these LUT configurations do not yield the desired weight dynamics (Figures 3, 4 and 6).

#### 3.3.3 Network benchmark: further constraints

In addition to the discretization of synaptic weights that has been analyzed so far, we also consider additional hardware constraints of the FACETS wafer-scale system (Section 2.6.3). This allows us to compare the effects of other hardware constraints to those of weight discretization.

First, we take into account a limited update controller frequency *v*_c_. Figure 7F shows that low frequencies (<1 Hz) distort the weight dynamics drastically and deteriorate the distinction between correlated and uncorrelated inputs. Ideally, a weight update would be performed whenever the spike pair accumulations cross the threshold (Figure 5A). However, these weight updates of frequency *v*_w_ are now limited to a time grid with frequency *v*_c_. The larger the latency between a threshold crossing and the arrival of the weight update controller, the more likely this threshold is exceeded. Hence, the weight update is underestimated and delayed. Low weight resolutions are less affected, because a high ratio *v*_c_/*v*_w_ reduces threshold overruns and hence distortions. This low resolution requires a high number of SSPs which in turn increases the threshold *a*_th_ (equation 3) and thus the weight update frequency *v*_w_.

Second, hardware-inspired synapses with the limitation to common reset lines cease to discriminate between correlated and uncorrelated input (Figure 7G, yellow and magenta traces). A crossing of the threshold by one spike pair accumulation resets the other (Figure 5) and suppresses its further weight updates, leading to underestimation of synapses with less correlated input.

To compensate for common resets we suggest ADCs that allow the comparison of spike pair accumulations to multiple thresholds. Nevertheless, ADCs compensate common resets only for high weight resolutions (Figure 7G). Again, for low weight resolutions and hence high numbers of SSPs fluctuations can not be taken into account (Figure 7G, gray values). This is the case for a 4-bit weight resolution, whereas a 8-bit weight resolution is high enough to resolve small fluctuations down to single SSPs (Figure 7G, cyan values). Each threshold has its own LUT configured with a number of SSPs that matches the dynamic range (Figure 3). The upper limit of *n* is chosen according to the results of Section 3.2. The update controller frequency is chosen to be low enough (*v*_c_ = 0.2 Hz) to enable all thresholds to be hit.

### 3.4 Hardware variations

**Figure 8:**
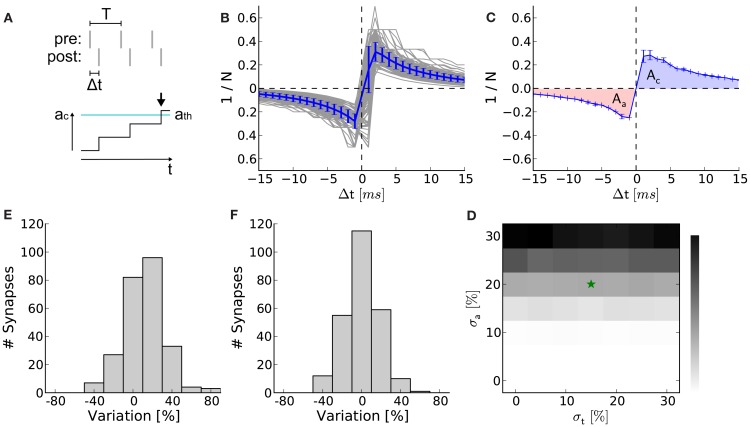
**Measurement of hardware synapse variations and their effects on learning in the neural network benchmark**. **(A)** Setup for recording STDP curves. At the top, spike trains of the pre- and postsynaptic neuron. Spike pairs with latency Δ*t* are repeated with frequency 1/*T*. At the bottom, a spike pair accumulation that crosses the threshold *a*_th_ (arrow). The inverse of the number of SSPs until crossing *a*_th_ (here *n* = 3) is plotted in **(B)**. **(B)** STDP curves of 252 hardware synapses within one synapse column (gray) and their mean with error (blue). A speed-up factor of 10^5^ is assumed. These curves correspond to *x*(Δ*t*) in equation (1), whereas *F*(*w*) is realized by the LUT. **(C)** One arbitrarily chosen STDP curve (over 5 trials) showing the areas for Δ*t* < 0 (*A*_a_ in red) and Δ*t* > 0 (*A*_c_ in blue). **(D)** Asymmetry between *A*_a_ and *A*_c_ within synapses (σ_a_ = 21%). **(E)** Variation of the absolute areas between synapses (σ_a_ = 17%). **(F)** The *p*-value (as in Figures 7E–G) in dependence on σ_a_ and σ_t_. The values for **(D,E)** are marked with an asterisk.

So far, we neglected production imperfections in real hardware systems. However, fixed pattern noise induced by these imperfections are a crucial limitation on the transistor level and may distort the functionality of the analog synapse circuit making higher weight resolutions unnecessary. The smaller and denser the transistors, the larger the discrepancies from their theoretical properties (Pelgrom et al., 1989). Using the protocol illustrated in Figure 8A we recorded STDP curves on the FACETS chip-based hardware system (Figures 8B,C; Section 2.7.1). Variations within (σ_a_) and between (σ_t_) individual synapses are shown as distributions in Figures 8D,E, both suggesting variations at around 20%. Both variations are incorporated into computer simulations of the network benchmark (Figure 7A; Section 2.7.2) to analyze their effects on synchrony detection. The *p*-value (as in Figures 7E–G) rises with increasing asymmetry within synapses, but is hardly affected by variations between synapses (Figure 8F).

## 4 Discussion

### 4.1 Configuration of STDP on discrete weights

In this study, we demonstrate generic strategies to configure STDP on discrete weights as, e.g. implemented in neuromorphic hardware systems. Resulting weight dynamics is critically dependent on the frequency of weight updates that has to be adjusted to the available weight resolution. Choosing a frequency within the dynamic range (Figure 3) is a prerequisite for the exploitation of discrete weight space ensuring proper weight dynamics. Analyses on long-term dynamics using Poisson-driven equilibrium weight distributions help to refine this choice (Figure 4). The obtained configuration space is similar to that of short-term dynamics, being the evolution of single synaptic weights (Figure 6). This similarity confirms the crucial impact of the LUT configuration on weight dynamics which is caused by rounding effects. Based on these results, we have chosen two example LUT configurations (*r* = 4 bits; *n* = 36 and *r* = 8 bits; *n* = 12) for further analysis, both realizable on the FACETS wafer-scale hardware system. High weight resolutions allow for higher frequencies of weight updates approximating the ideal model, occasionally requiring several spike pairs to evoke a weight update. Correspondingly, in associative pairing literature, a minimal number of associations is required to detect functional changes (expressed by the spiking or postsynaptic potential response) and varies from studies to studies from a few to several tens (Cassenaer and Laurent, 2007, 2012).

Discretization not only affects the accuracy of weights, but also broadens their equilibrium weight distributions (Figure 4), which are actually shown to be narrow in large-scale neural networks (Morrison et al., 2007). Furthermore, this broadening can distort the functionality of neural networks, e.g. it deteriorates the distinction between the two groups of weights (of synapses originating from the correlated or uncorrelated population) within the network benchmark (compare Figures 7C,D). On the other hand, weight discretization can also be advantageous for synchrony detection, if, e.g. groups of weights separate due to large step sizes between neighboring discrete weights (compare red and green in Figure 7E).

In summary, these analyses of STDP on discrete weights are necessary for obtaining appropriate configurations for a variety of STDP models and weight resolutions.

### 4.2 4-bit weight resolution

Simulations of the network benchmark show that a 4-bit weight resolution is sufficient to detect synchronous presynaptic firing significantly (Figure 7). Groups of synapses receiving correlated input strengthen and in turn increase the probability of synchronous presynaptic activity to elicit postsynaptic spikes as compared to static synapses (Figure 7B). Thus, the weight distribution within the network reflects synchrony within sub-populations of presynaptic neurons. Increasing the weight resolution causes both weight distributions, for the correlated and uncorrelated input, to narrow and separate from each other. Consequently, an 8-bit resolution is sufficient to reproduce the *p*-values of continuous weights with floating point precision (corresponds to discrete weights with *r* = 64 bits, Figure 7E). This resolution requires the combination of two hardware synapses and is under development (Schemmel et al., 2010). On the other hand, increasing the weight resolution, but retaining the frequency of weight updates (number of SSPs), results in weight distributions of comparable width and consequently does not improve the *p*-values significantly (Figure 7E).

Other neuromorphic hardware systems implement bistable synapses corresponding to a 1-bit weight resolution (Badoni et al., 2006; Indiveri et al., 2010). Bistable synapse models are shown to be sufficient for memory formation (Amit and Fusi, 1994; Fusi et al., 2005; Brader et al., 2007; Clopath et al., 2008). However, these models do not only employ spike-timings (Levy and Steward, 1983; Markram, 2006; Mu and Poo, 2006; Cassenaer and Laurent, 2007; Bill et al., 2010), but also read the postsynaptic membrane potential (Sjöström et al., 2001; Trachtenberg et al., 2002) requiring additional hardware resources. So far, there is no consensus of a general synapse model, and neuromorphic hardware systems are mostly limited to only subclasses of these models.

Studies on weight discretization are not limited to the FACETS hardware systems only, but are applicable to other backends for neural network simulations. For example, our results can be applied to the fully digital neuromorphic hardware system described by Jin et al. (2010b), who also report STDP with a reduced weight resolution. Furthermore, weight discretization may be a further approach to reduce memory consumption of “classical” neural simulators.

### 4.3 Further hardware constraints

In addition to a limited weight resolution, we have studied further constraints of the current FACETS wafer-scale hardware system with the network benchmark.

A limited update controller frequency implying a minimum time interval between subsequent weight updates does not affect the *p*-values down to a critical frequency *v*_c_ ≈ 1 Hz (Figure 7F). The update controller frequency decreases linearly with the number of hardware synapses enabled for STDP. Assuming a hardware acceleration factor of 10^3^ all synapses can be enabled for STDP staying below this critical frequency. However, the number of STDP synapses should be decreased if a higher update controller frequency is required, e.g. for a configuration with an 8-bit weight resolution and a small number of SSPs.

Common resets of spike pair accumulations reduce synapse chip resources by requiring one instead of two reset lines, but suppress synaptic depression and bias the weight evolution toward potentiation. This is due to the feed-forward network architecture, in which causal relationships between pre- and postsynaptic spikes are more likely than anti-causal ones. Long periods of accumulation (large numbers of SSPs) lower the probability of synaptic depression. Hence, all weights tend to saturate at the maximum weight value impeding a distinction between both populations of synapses within the network benchmark (Figure 7G). The probability of synaptic depression can be increased by high weight update frequencies (small numbers of SSPs) shortening the accumulation periods (equation 3) and subsequently approaching the behavior of independent resets. However, high weight update frequencies require high weight resolutions and thus high update controller frequencies, which decreases the number of available synapses enabled for STDP.

As a compensation for common resets, we suggest that the single spike pair accumulation threshold is expanded to multiple thresholds implemented as ADCs. In comparison to synapses with common resets, ADCs improve *p*-values significantly only for an 8-bit weight resolutions (Figure 7G, compare cyan to magenta values). However, the combination of two 4-bit hardware synapses allows to mimic independent resets and hence yields *p*-values comparable to 8-bit synapses using ADCs (Figure 7G, compare red to cyan values). Mimicking independent resets is under development for the FACETS wafer-scale hardware system. Each of the two combined synapses will be configured to accumulate only either causal or anti-causal spike pairs, while both synapses are updated in a common process. This requires only minor hardware design changes within the weight update controller and should be preferred to more expensive changes for realizing ADCs. The implementation of real second reset lines is not possible without major hardware design changes, but is considered for future chip revisions.

Benchmark simulations incorporating the measured variations within and between synapse circuits due to production imperfections result in *p*-values worse (higher) than for a 4-bit weight resolution (compare asterisk in Figure 8F to red value for *c* = 0.025 in Figure 7E). Consequently, a 4-bit weight resolution is sufficient for the current implementation of the measurement and accumulation circuits. We suppose that the isolatedly analyzed effects of production imperfections and weight discretization add up and limit the best possible *p*-value of each other. Analysis on combinations of hardware restrictions would allow to quantify how their effects add up and are considered for further studies. However, hardware variations can also be considered as a limitation on the transistor level making higher weight resolutions unnecessary.

**Figure 9:**
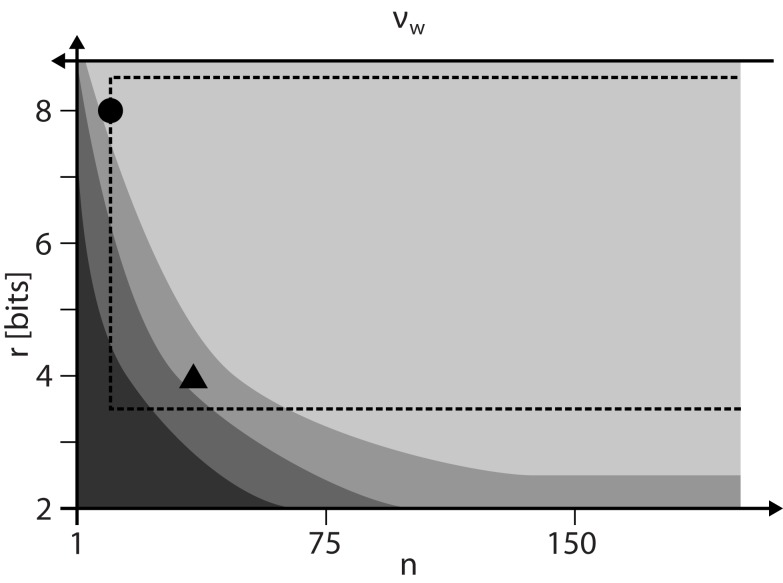
**The configuration space of STDP on discrete weights spanned by the weight resolution *r* and the number *n* of SSPs that is inversely proportional to the weight update frequency *v*_w_**. The darkest gray area depicts the configurations with dead discrete weights (Figure 3). The lower limits of configurations for proper equilibrium weight distributions (Figure 4) and single synapse dynamics (Figure 6) are shown with brighter shades. The dashed rectangle marks configurations realizable by the FACETS wafer-scale hardware system (assuming an acceleration factor of 10^3^, all synapses enabled for STDP and SSPs applied with 10 Hz). The working points for a 4-bit (*n* = 36) and 8-bit (*n* = 12) weight resolution are highlighted as a triangle and circle, respectively.

Figure 9 summarizes the results on how to configure STDP on discrete weights. For a given weight resolution *r* the number *n* of SSPs has to be chosen as low as possible to allow for high weight update frequencies *v*_w_. However, *n* must be high enough to ensure STDP dynamics comparable to continuous weights (lightest gray shaded area) and to stay within the configuration space realizable by the FACETS wafer-scale hardware system. The hardware system limits the update controller frequency *v*_c_ and hence distorts STDP especially for low *n*.

### 4.4 Outlook

Currently, STDP in neuromorphic hardware systems is enabled for only 10 to few 10,000 synapses in real-time (Arthur and Boahen, 2006; Zou et al., 2006; Daouzli et al., 2008; Ramakrishnan et al., 2011). Large-scale systems do not implement long-term plasticity (Merolla and Boahen, 2006; Vogels et al., 2011) or operate in real-time only (Jin et al., 2010a). Enabling a large-scale (over 4·10^7^ synapses) and highly accelerated neuromorphic hardware system (the FACETS wafer-scale hardware system) with configurable STDP requires trade-offs between number and size of synapses, which raises constraints in their implementation (Schemmel et al., 2006, 2010). Table 5 summarizes these trade-offs and gives an impression about the hardware costs and effects on STDP.

**Table 5:** **Possible design modifications of hardware synapses, their reduction in terms of required chip resources and their effects on STDP**.

Modification	Resource reduction	Effect on STDP
Global weight update controller	+++	Latency between synapse processings; spike pair accumulations necessary
Analog measurement of spike-timing-dependence	++	Analog measurements are affected by production imperfections
Reduced spike pairing scheme	++	n.a.
Decreased weight resolution	++	Loss in synapse dynamics and competition; large weight steps require spike pair accumulations
Operation frequency *v*_c_ of the weight update controller (overall frequency could be increased by implementing multiple controllers)	++	Threshold over-shootings distorts synchrony detection
Common reset line	+	No synchrony detection possible
LUTs (compared to arithmetic operations)	+	None
ADCs as compensation for common resets	−	No significant compensation in case of 4-bit synapses

*These modifications are listed by their resource reduction in descending order inspired by the FACETS wafer-scale hardware system and its production process. A larger reduction of chip resources allows more synapses on a single chip*.

**Table 6:** **Applied hardware parameters**.

Parameter	Description	Value
*V*_clrc_	Amount of charge that will be accumulated on the capacitor *C*_1_ (Schemmel et al., 2006) in case of causal spike time correlations, corresponds to *x*(Δ*t*)	0.90 V
*V*_clra_	See *V*_clrc_, but for the anti-causal circuit	0.94 V
*V*_ctlow_	Lower spike pair accumulation threshold	0.85 V
*V*_cthigh_	Higher spike pair accumulation threshold	1.0 V
*Adjdel*	Adjustable delay between the pre- and postsynaptic spike	2.5 μA
*V*_m_	Parameter to stretch the STDP time constant τ_STDP_	0.0 V
*I*_bcorreadb_	Bias current that influences timing issues during read outs	2.0 μA
*drvI*_rise_	Rise time of synaptic conductance	1.0 V
*drvI*_fall_	Fall time of synaptic conductance	1.0 V
*V*_start_	Start value of synaptic conductance, need for small rise times	0.25 V
*drvI*_out_	Maximum value of synaptic conductance, corresponds to *g*_max_	Variable

*The difference *V*_cthigh_ − *V*_ctlow_ corresponds to the threshold *a*_th_. All data is recorded with the FACETS chip-based hardware system using chip number 444 and synapse column 4*.

**Table 7:** **Model description of the network benchmark using the reference synapse model**. After Nordlie et al. (2009).

**A: MODEL SUMMARY**		
Populations	Three: uncorrelated input (U), correlated input (C), target (T)	
Topology	Feed-forward	
Connectivity	All-to-one	
Neuron model	Leaky integrate-and-fire, fixed voltage threshold, fixed absolute refractory period (voltage clamp)	
Synapse model	Exponential-shaped postsynaptic conductances	
Plasticity	Intermediate Gütig spike-timing dependent plasticity	
Input	Fixed-rate Poisson (for U) and multiple interaction process (for C) spike trains	
Measurements	Synaptic weights	
**B: POPULATIONS**		
Name	Elements	Population size
U	Parrot neurons	*N*_u_
C	Parrot neurons	*N*_c_
T	IAF neurons	*N*_T_
**C: CONNECTIVITY**		
Source	Target	Pattern
U	T	All-to-all, uniformly distributed initial weights *w*, STDP, delay *d*
**D: NEURON AND SYNAPSE MODEL**		
Name	IAF neuron	
Type	Leaky integrate-and-fire, exponential-shaped synaptic conductances	
Sub-threshold dynamics	*C*_m_ d*V*/d*t* = *g_L_* (*E_L_* − *V*) + *g*(*t*) (*E*_e_ − *V*) if *t* > t* + τ_ref_ *V*(*t*) = *V*_reset_ else *g*(*t*) = *wg*_max_ exp(−*t*/τ_syn_)	
Spiking	If *V*(*t*−) < þeta ∧ *V*(*t*+) ≥ θ 1. Set t* = *t*, 2. Emit spike with time stamp t*	
Name	Parrot neuron	
Type	Repeats input spikes with delay *d*	
**E: PLASTICITY**		
Name	Intermediate Gütig STDP	
Spike pairing scheme	Reduced symmetric nearest-neighbor	
Weight dynamics	δw(w,Δt)=F(w)x(Δt)	
	*x*(Δ*t*) = exp(−|Δ*t*|/τ_STDP_)	
	*F*(*w*) = λ(1 − *w*)^μ^ if Δ*t* > 0	
	*F*(*w*) = −λα*w*^μ^ if Δ*t* < 0	
**F: INPUT**		
Type	Target	Description
Poisson generators	U	Independent Poisson spike trains with firing rate *ρ*
MIP generators	C	Spike trains with correlation *c* and firing rate *ρ*
**G: MEASUREMENTS**		
evolution and final distribution of all synaptic weights		

*For details about the hardware-inspired synapse model see Section* 2.6.1.

**Table 8:** **Parameter specification**.

Name	Value	Description
**B: POPULATIONS**		
*N*_u_	10	Number of neurons in uncorrelated input population
*N*_c_	10	Number of neurons in correlated input population
*N*_T_	1	Number of neurons in target population
**C: CONNECTIVITY**		
*w*	Uniformly distributed over [0, 1]	Number of neurons in uncorrelated input population
*d*	0.1 ms	Synaptic transmission delays
**D: NEURON AND SYNAPSE MODEL**		
*C*_m_	250 pF	Membrane capacity
*g*_L_	16.6667 nS	Leakage conductance
*E*_L_	−70 mV	Leakage reversal potential
θ	−55 mV	Fixed firing threshold
*V*_reset_	−60 mV	Reset potential
τ_ref_	2 ms	Absolute refractory period
*E*_e_	0 mV	Excitatory reversal potential
*g*_max_	100 nS	Postsynaptic maximum conductance
τ_syn_	0.2 ms	Postsynaptic conductance time constant
**E: PLASTICITY**		
α	1.05	Asymmetry
λ	0.005	learning rate
μ	0.4	Exponent
τ_STDP_	20 ms	STDP time constant
**F: INPUT**		
ρ	7.2 Hz	Firing rate
*c*	[0.005, 0.05]	Pair-wise correlation between spike trains

*The categories refer to the model description in Table 7*.

In this study, we introduced novel analysis tools allowing the investigation of hardware constraints and therefore verifying and improving the hardware design without the need for expensive and time-consuming prototyping. Ideally, this validation process should be shifted to an earlier stage of hardware design combining the expertise from Computational Neuroscience and Neuromorphic Engineering, as, e.g. published by Linares-Barranco et al. (2011). This kind of research is crucial for researchers to use and understand research executed on neuromorphic hardware systems and thereby transform it into a tool substituting von Neumann computers in Computational Neuroscience. Brüderle et al. (2011) report the development of a *virtual hardware*, a simulation tool replicating the functionality and configuration space of the entire FACETS wafer-scale hardware system. This tool will allow further analyses on hardware constraints, e.g. in the communication infrastructure and configuration space.

The presented results verify the current implementation of the FACETS wafer-scale hardware system in terms of balance between weight resolution, update controller frequency and circuit variations. Further improvement of the existing hardware implementation would require improvements of all aspects. The only substantial bottleneck has been identified to be common resets, already leading to design improvements of the wafer-scale system.

Although all presented studies refer to the intermediate Gütig STDP model, any other STDP model relying on equation (1) and an exponentially decaying time-dependence can be investigated with the existing software tools in a generic way, e.g. those models listed in Table 1. In contrast to the fixed exponential time-dependence implemented as analog circuits in the FACETS wafer-scale hardware system, the weight-dependence is freely programmable and stored in a LUT.

Ideally, a high resolution in the weight range of highest plausibility is requested, a high *effective resolution*. Bounded STDP models (e.g. the intermediate Gütig STDP model applied in this study) are well suited for a 4-bit weight resolution and allow a linear mapping of continuous to discrete weights. A 4-bit weight resolution causes large weight updates and hence broadens the weight distribution spanning the whole weight range. This results in a high effective resolution. On the other hand, unbounded STDP models (e.g. the power law and van Rossum STDP models) have long tails toward high weights. Cutting the tail by only mapping low weights to discrete weights would increase the frequency of the highest discrete weight. A possible solution is a non-linear mapping of continuous to discrete weights – large differences between high discrete weights and small differences between low discrete weights. However, a variable distance between discrete weights would require more hardware efforts.

An all-to-all spike pairing scheme applied to the reference synapses within the network benchmark results in *p*-values worse (higher) than for synapses implementing a reduced symmetric nearest-neighbor spike pairing scheme (not shown, but comparable to 4-bit discrete weights in Figure 7E, see red values). Detailed analyses on different spike pairing schemes could be investigated in further studies.

As a next step, our hardware synapse model can replace the regular STDP synapses in simulations of established neural networks, to test their robustness and applicability for physical emulation in the FACETS wafer-scale hardware system. The synapse model is available in the following NEST release and can easily be applied to NEST or PyNN network descriptions. If neural networks, or modifications of them, qualitatively reproduce the simulation, they can be applied to the hardware system, with which similar results can be expected. Thus, the presented simulation tools allow beforehand modifications of network architectures to ensure the compatibility with the hardware system.

With respect to more complex long-term plasticity models, the hardware system is currently being extended by a programmable microprocessor that is in control of all weight modifications. This processor allows to combine synapse rows in order to compensate for common resets. With possible access to further neuron or network properties the processor would allow for more complex plasticity rules as, e.g. those of Clopath et al. (2008) and Vogelstein et al. (2007). Even modifications of multiple neurons are feasible, a phenomenon observed in experiments with neuromodulators (Eckhorn et al., 1990; Itti and Koch, 2001; Reynolds and Wickens, 2002; Shmuel et al., 2005). Nevertheless, more experimental data and consensus about neuromodulator models and their applications are required to further customize the processor. New hardware revisions are rather expensive and consequently should only cover established models that are prepared for hardware implementation by dedicated studies.

This presented evaluation of the FACETS wafer-scale hardware system is meant to encourage neuroscientists to benefit from neuromorphic hardware without leaving their environment in terms of neuron, synapse and network models. We further endorse that, toward an efficient exploitation of hardware resources, the design of synapse models will be influenced by hardware implementations rather than only by their mathematical treatability (e.g. Badoni et al., 2006).

## Conflict of Interest Statement

The authors declare that the research was conducted in the absence of any commercial or financial relationships that could be construed as a potential conflict of interest.

## Footnotes

^1^Fast Analog Computing with Emergent Transient States

^2^One weight update controller for all 256 neurons with 224 synapses each.

## A Appendix

### A.1 Analytical distributions

Weight evolutions can be described by asymmetric Markov processes with boundary conditions. Following van Rossum et al. (2000), the weight distribution *P*(*w*) can be expressed by a Taylor expansion of the underlying master equation

$$\begin{array}{rcll} \frac{\partial P\left(w,t\right)}{\partial t} & =-p_{\text{d}}P\left(w,t\right)-p_{\text{p}}P\left(w,t\right)+p_{\text{d}}P\left(w+\Delta w_{\text{d}},t\right) & & \\ & \;+p_{\text{p}}P\left(w-\Delta w_{\text{p}},t\right). & & \text{(A1)} \end{array}$$

In contrast to van Rossum et al. (2000), this study defines a weight step Δ*w* by a sequence of *n* weight updates δ*w* as described by equation (1). Hence the weight steps Δ*w* can be written as Δ*w*_d_(*w*) = (*w* + *F*_−_(*w*))*_n_* − *w* and Δ*w*_p_(*w*) = (*w* + *F*_+_(*w*))*_n_* − *w*, where *f*(*w*)*_n_* is the *n*-th recursive evaluation of *f*(*w*).

According to van Rossum et al. (2000) this Taylor expansion results in the Fokker–Planck equation

(A2) $$\frac{\partial P\left(w,t\right)}{\partial t}=-\frac{\partial }{\partial w}\left[A\left(w\right)P\left(w,t\right)\right]+\frac{1}{2}\frac{\partial^{2}}{\partial w^{2}}\left[B\left(w\right)P\left(w,t\right)\right]$$

with jump moments *A*(*w*) = *p*_d_Δ*w*_d_(*w*) + *p*_p_Δ*w*_p_(*w*) and *B*(*w*) = *p*_d_Δ*w*_d_(*w*)^2^ + *p*_p_Δ*w*_p_(*w*)^2^, which has the following solution for reflecting boundary conditions (Gardiner, 2009):

(A3) $$P\left(w\right)=\frac{N}{B\left(w\right)}\exp\left[2\int_{0}^{w}\;\frac{A\left(w^{\prime }\right)}{B\left(w^{\prime }\right)}dw^{\prime }\right],$$

with *N* as a normalization factor. For small *n* this equation can be solved analytically, but is integrated numerically to cover also large *n*.

However, this analytical approach fails, because the Taylor expansion in combination with the boundary conditions does not hold for large *n* (absorbing boundary conditions do not improve the results).

### A.2 STDP in the FACETS chip-based hardware system

The STDP mechanism of the FACETS chip-based hardware system differs from that of the FACETS wafer-scale hardware system as follows. The major difference is the comparison of spike pair accumulations with thresholds. The wafer-scale system analyzed in this study compares both spike pair accumulations with a threshold (the threshold can be set independently for both accumulations, but they are assumed to be equal in this study). An weight update is performed if a single accumulation crosses this threshold. In contrast, the chip-based system used for all measurements subtracts both spike pair accumulations and compares the absolute value of their difference |*a*_c_ − *a*_a_| with a single threshold. If this threshold is crossed, the sign of the difference between the spike pair accumulations sig (*a*_c_ − *a*_a_) determines, whether the causal or anti-causal accumulation prevails and the weight is updated accordingly. However, this difference between both hardware systems can be neglected, because both STDP mechanisms are identical if exclusively causal or anti-causal spike pairs are accumulated. This is the case for the measurement protocol of STDP curves.

### A.3 Generating spike pairs in hardware

Spike pairs in the FACETS chip-based hardware system are generated as follows. Presynaptic spike times can be set precisely, whereas postsynaptic spikes need to be triggered by presynaptic input. Therefore, a presynaptic spike (via the measured synapse) and *m* trigger spikes (eliciting a postsynaptic spike) are fed into a single neuron occupying *m* + 1 synapses. The synaptic weights as well as the synapse driver strengths of the trigger synapses are proportional to the synaptic peak conductance and are adjusted in such a way that a single postsynaptic spike is evoked. The highest reliability of spike times within a hardware run and between runs is achieved for *m* = 4 trigger synapses (not shown here). The synapse driver strength is set to the intermediate value between the limiting case of no and multiple postsynaptic spikes evoked by one trigger only. The synaptic weight of the measured synapse is set to zero and consequently the measured synapse has no influence on the elicitation of postsynaptic spikes.
